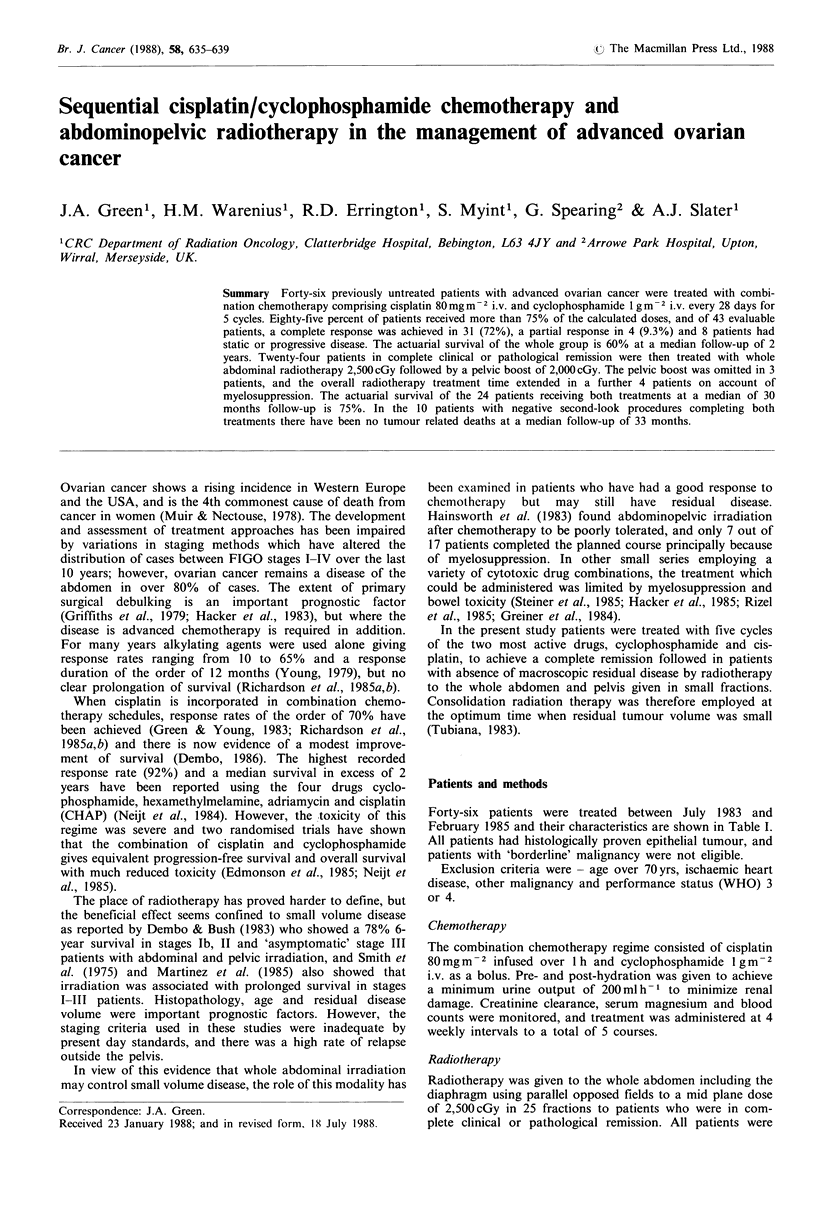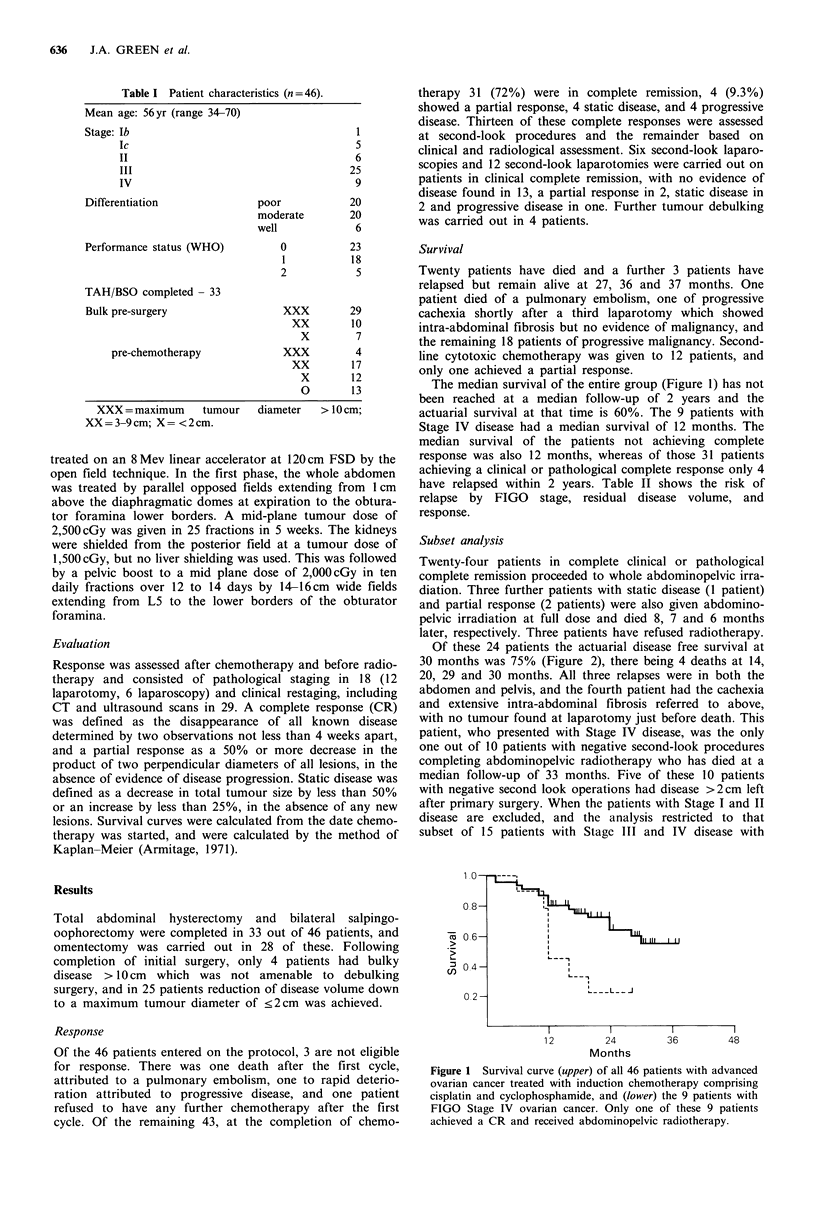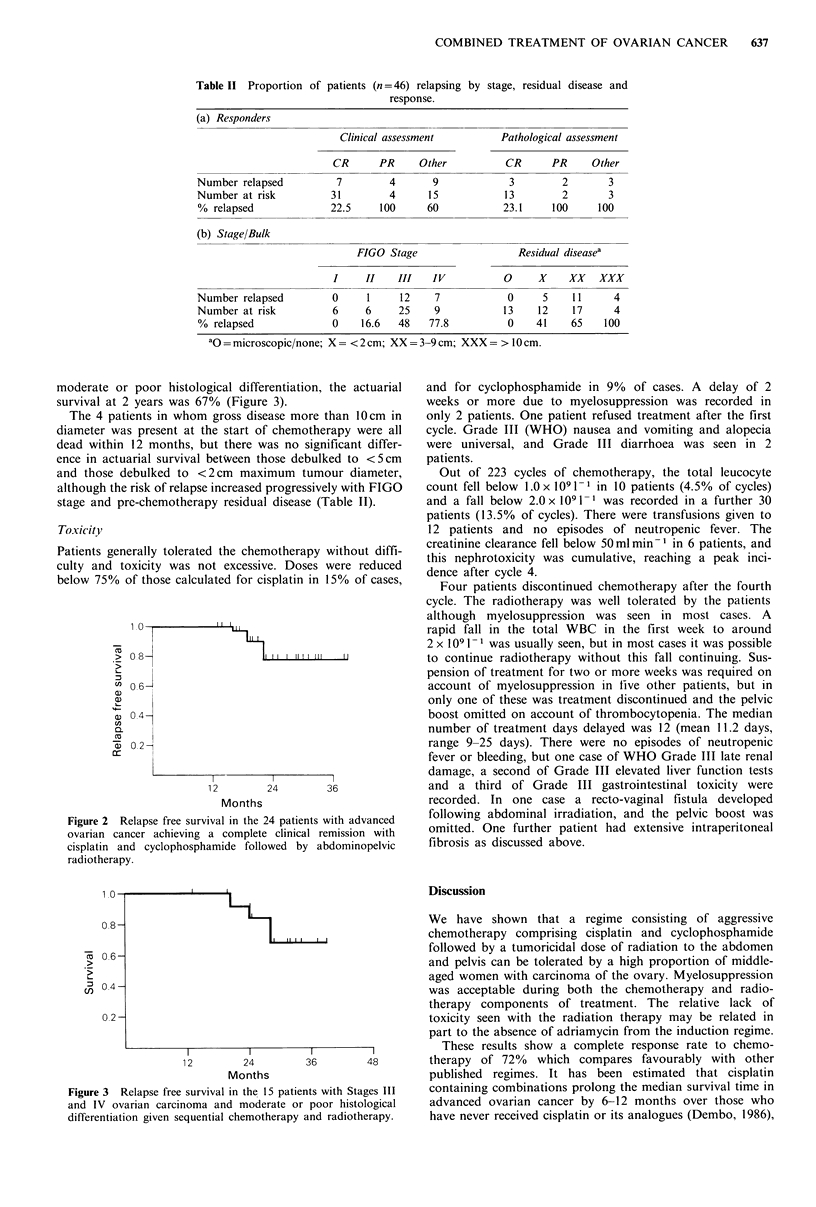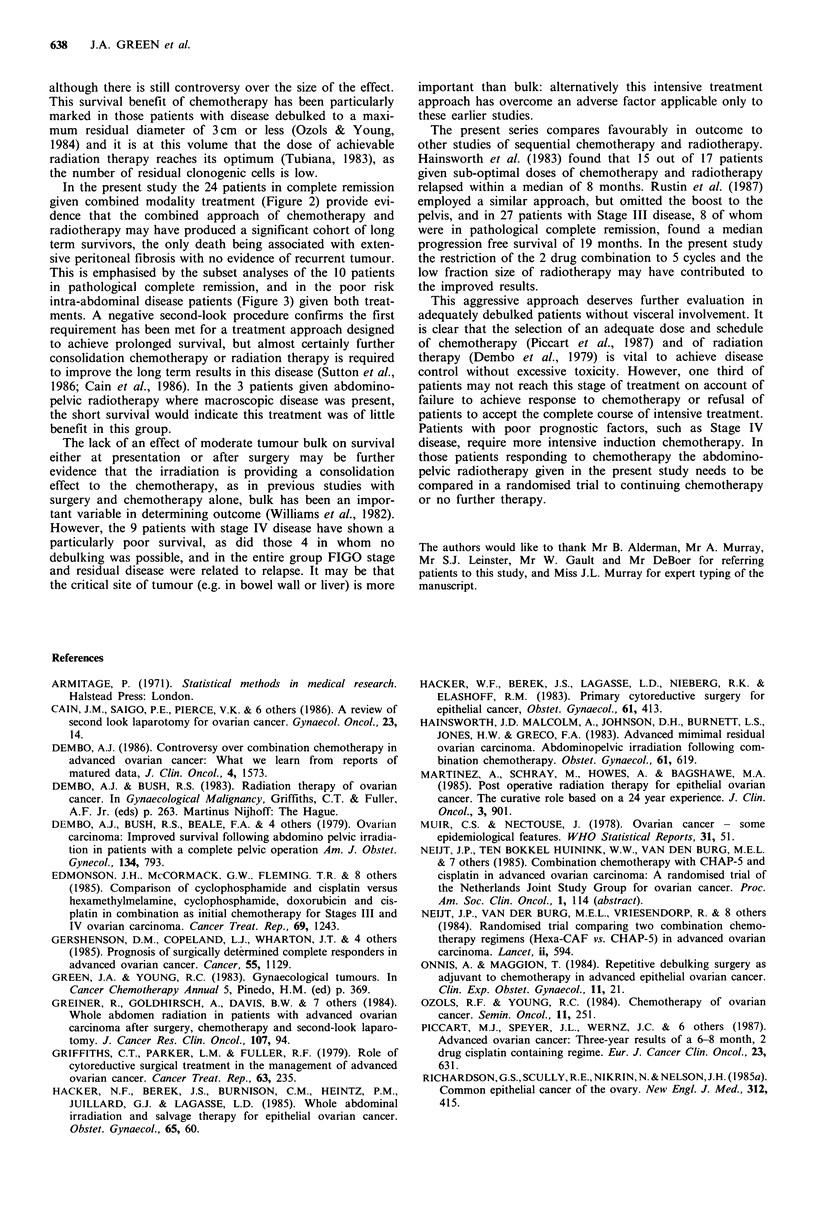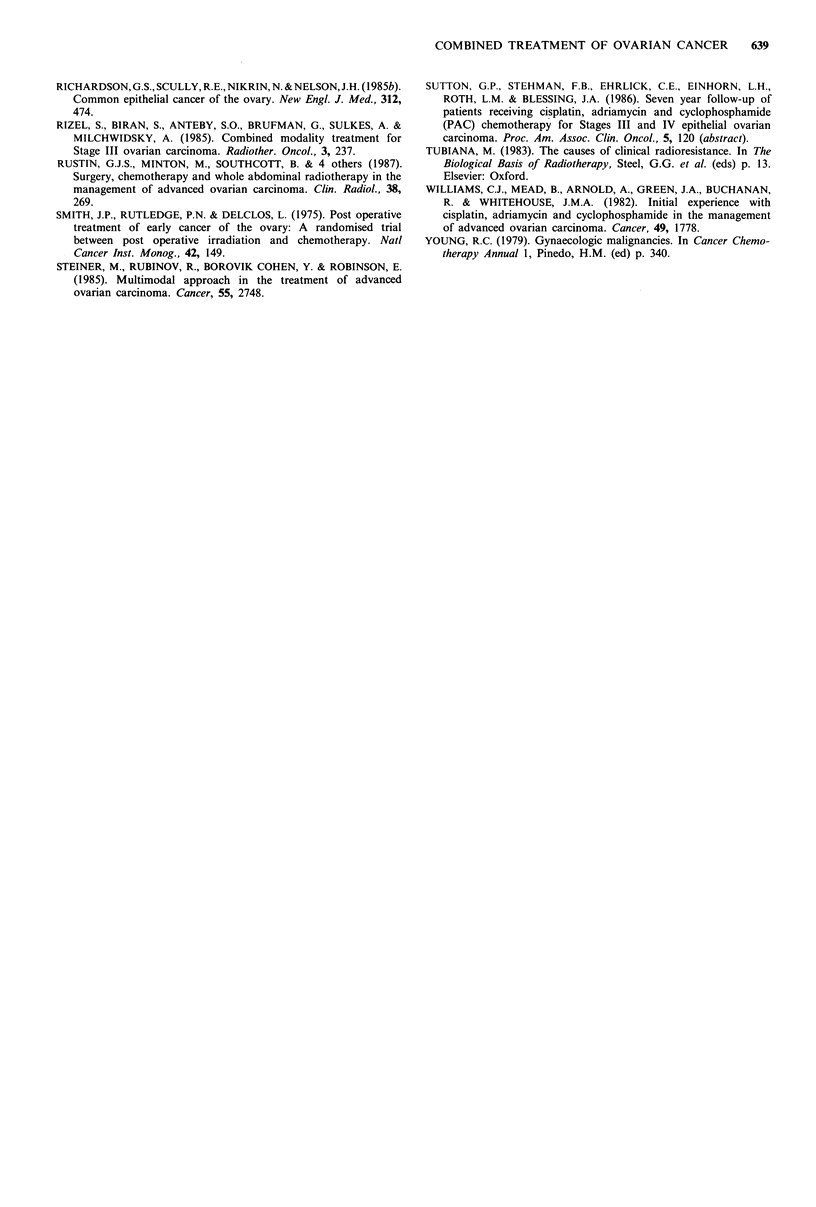# Sequential cisplatin/cyclophosphamide chemotherapy and abdominopelvic radiotherapy in the management of advanced ovarian cancer.

**DOI:** 10.1038/bjc.1988.275

**Published:** 1988-11

**Authors:** J. A. Green, H. M. Warenius, R. D. Errington, S. Myint, G. Spearing, A. J. Slater

**Affiliations:** CRC Department of Radiation Oncology, Clatterbridge Hospital, Bebington, UK.

## Abstract

Forty-six previously untreated patients with advanced ovarian cancer were treated with combination chemotherapy comprising cisplatin 80 mg m-2 i.v. and cyclophosphamide 1 gm-2 i.v. every 28 days for 5 cycles. Eighty-five percent of patients received more than 75% of the calculated doses, and of 43 evaluable patients, a complete response was achieved in 31 (72%), a partial response in 4 (9.3%) and 8 patients had static or progressive disease. The actuarial survival of the whole group is 60% at a median follow-up of 2 years. Twenty-four patients in complete clinical or pathological remission were then treated with whole abdominal radiotherapy 2,500 cGy followed by a pelvic boost of 2,000 cGy. The pelvic boost was omitted in 3 patients, and the overall radiotherapy treatment time extended in a further 4 patients on account of myelosuppression. The actuarial survival of the 24 patients receiving both treatments at a median of 30 months follow-up is 75%. In the 10 patients with negative second-look procedures completing both treatments there have been no tumour related deaths at a median follow-up of 33 months.


Br. J. Cancer (1988), 58, 635–639 © The Macmillan Press Ltd., 1988

### Sequential cisplatin/cyclophosphamide chemotherapy and abdominopelvic radiotherapy in the management of advanced ovarian cancer

J.A. Green[1], H.M. Warenius[1], R.D. Errington[1], S. Myint[1], G. Spearing[2] & A.J. Slater[1]

[1]*CRC Department of Radiation Oncology, Clatterbridge Hospital, Bebington, L63 4JY and* [2]*Arrowe Park Hospital, Upton, Wirral, Merseyside, UK.*

**Summary** Forty-six previously untreated patients with advanced ovarian cancer were treated with combination chemotherapy comprising cisplatin 80 mg m$^{-2}$ i.v. and cyclophosphamide 1 g m$^{-2}$ i.v. every 28 days for 5 cycles. Eighty-five percent of patients received more than 75% of the calculated doses, and of 43 evaluable patients, a complete response was achieved in 31 (72%), a partial response in 4 (9.3%) and 8 patients had static or progressive disease. The actuarial survival of the whole group is 60% at a median follow-up of 2 years. Twenty-four patients in complete clinical or pathological remission were then treated with whole abdominal radiotherapy 2,500 cGy followed by a pelvic boost of 2,000 cGy. The pelvic boost was omitted in 3 patients, and the overall radiotherapy treatment time extended in a further 4 patients on account of myelosuppression. The actuarial survival of the 24 patients receiving both treatments at a median of 30 months follow-up is 75%. In the 10 patients with negative second-look procedures completing both treatments there have been no tumour related deaths at a median follow-up of 33 months.

Ovarian cancer shows a rising incidence in Western Europe and the USA, and is the 4th commonest cause of death from cancer in women (Muir & Nectouse, 1978). The development and assessment of treatment approaches has been impaired by variations in staging methods which have altered the distribution of cases between FIGO stages I–IV over the last 10 years; however, ovarian cancer remains a disease of the abdomen in over 80% of cases. The extent of primary surgical debulking is an important prognostic factor (Griffiths *et al.*, 1979; Hacker *et al.*, 1983), but where the disease is advanced chemotherapy is required in addition. For many years alkylating agents were used alone giving response rates ranging from 10 to 65% and a response duration of the order of 12 months (Young, 1979), but no clear prolongation of survival (Richardson *et al.*, 1985*a,b*).

When cisplatin is incorporated in combination chemotherapy schedules, response rates of the order of 70% have been achieved (Green & Young, 1983; Richardson *et al.*, 1985*a,b*) and there is now evidence of a modest improvement of survival (Dembo, 1986). The highest recorded response rate (92%) and a median survival in excess of 2 years have been reported using the four drugs cyclophosphamide, hexamethylmelamine, adriamycin and cisplatin (CHAP) (Neijt *et al.*, 1984). However, the toxicity of this regime was severe and two randomised trials have shown that the combination of cisplatin and cyclophosphamide gives equivalent progression-free survival and overall survival with much reduced toxicity (Edmonson *et al.*, 1985; Neijt *et al.*, 1985).

The place of radiotherapy has proved harder to define, but the beneficial effect seems confined to small volume disease as reported by Dembo & Bush (1983) who showed a 78% 6-year survival in stages Ib, II and 'asymptomatic' stage III patients with abdominal and pelvic irradiation, and Smith *et al.* (1975) and Martinez *et al.* (1985) also showed that irradiation was associated with prolonged survival in stages I–III patients. Histopathology, age and residual disease volume were important prognostic factors. However, the staging criteria used in these studies were inadequate by present day standards, and there was a high rate of relapse outside the pelvis.

In view of this evidence that whole abdominal irradiation may control small volume disease, the role of this modality has been examined in patients who have had a good response to chemotherapy but may still have residual disease. Hainsworth *et al.* (1983) found abdominopelvic irradiation after chemotherapy to be poorly tolerated, and only 7 out of 17 patients completed the planned course principally because of myelosuppression. In other small series employing a variety of cytotoxic drug combinations, the treatment which could be administered was limited by myelosuppression and bowel toxicity (Steiner *et al.*, 1985; Hacker *et al.*, 1985; Rizel *et al.*, 1985; Greiner *et al.*, 1984).

In the present study patients were treated with five cycles of the two most active drugs, cyclophosphamide and cisplatin, to achieve a complete remission followed in patients with absence of macroscopic residual disease by radiotherapy to the whole abdomen and pelvis given in small fractions. Consolidation radiation therapy was therefore employed at the optimum time when residual tumour volume was small (Tubiana, 1983).

### Patients and methods

Forty-six patients were treated between July 1983 and February 1985 and their characteristics are shown in Table I. All patients had histologically proven epithelial tumour, and patients with 'borderline' malignancy were not eligible.

Exclusion criteria were – age over 70 yrs, ischaemic heart disease, other malignancy and performance status (WHO) 3 or 4.

### *Chemotherapy*

The combination chemotherapy regime consisted of cisplatin 80 mg m$^{-2}$ infused over 1 h and cyclophosphamide 1 g m$^{-2}$ i.v. as a bolus. Pre- and post-hydration was given to achieve a minimum urine output of 200 ml h$^{-1}$ to minimize renal damage. Creatinine clearance, serum magnesium and blood counts were monitored, and treatment was administered at 4 weekly intervals to a total of 5 courses.

### *Radiotherapy*

Radiotherapy was given to the whole abdomen including the diaphragm using parallel opposed fields to a mid plane dose of 2,500 cGy in 25 fractions to patients who were in complete clinical or pathological remission. All patients were

Correspondence: J.A. Green.
Received 23 January 1988; and in revised form, 18 July 1988.

**Table I** Patient characteristics ($n = 46$).

| | | |
|---|---|---|
| Mean age: 56 yr (range 34–70) | | |
| Stage: I*b* | | 1 |
| I*c* | | 5 |
| II | | 6 |
| III | | 25 |
| IV | | 9 |
| Differentiation | poor | 20 |
| | moderate | 20 |
| | well | 6 |
| Performance status (WHO) | 0 | 23 |
| | 1 | 18 |
| | 2 | 5 |
| TAH/BSO completed – 33 | | |
| Bulk pre-surgery | XXX | 29 |
| | XX | 10 |
| | X | 7 |
| pre-chemotherapy | XXX | 4 |
| | XX | 17 |
| | X | 12 |
| | O | 13 |

XXX = maximum tumour diameter > 10 cm; XX = 3–9 cm; X = < 2 cm.

treated on an 8 Mev linear accelerator at 120 cm FSD by the open field technique. In the first phase, the whole abdomen was treated by parallel opposed fields extending from 1 cm above the diaphragmatic domes at expiration to the obturator foramina lower borders. A mid-plane tumour dose of 2,500 cGy was given in 25 fractions in 5 weeks. The kidneys were shielded from the posterior field at a tumour dose of 1,500 cGy, but no liver shielding was used. This was followed by a pelvic boost to a mid plane dose of 2,000 cGy in ten daily fractions over 12 to 14 days by 14–16 cm wide fields extending from L5 to the lower borders of the obturator foramina.

*Evaluation*

Response was assessed after chemotherapy and before radiotherapy and consisted of pathological staging in 18 (12 laparotomy, 6 laparoscopy) and clinical restaging, including CT and ultrasound scans in 29. A complete response (CR) was defined as the disappearance of all known disease determined by two observations not less than 4 weeks apart, and a partial response as a 50% or more decrease in the product of two perpendicular diameters of all lesions, in the absence of evidence of disease progression. Static disease was defined as a decrease in total tumour size by less than 50% or an increase by less than 25%, in the absence of any new lesions. Survival curves were calculated from the date chemotherapy was started, and were calculated by the method of Kaplan–Meier (Armitage, 1971).

### Results

Total abdominal hysterectomy and bilateral salpingo-oophorectomy were completed in 33 out of 46 patients, and omentectomy was carried out in 28 of these. Following completion of initial surgery, only 4 patients had bulky disease > 10 cm which was not amenable to debulking surgery, and in 25 patients reduction of disease volume down to a maximum tumour diameter of ≤2 cm was achieved.

*Response*

Of the 46 patients entered on the protocol, 3 are not eligible for response. There was one death after the first cycle, attributed to a pulmonary embolism, one to rapid deterioration attributed to progressive disease, and one patient refused to have any further chemotherapy after the first cycle. Of the remaining 43, at the completion of chemotherapy 31 (72%) were in complete remission, 4 (9.3%) showed a partial response, 4 static disease, and 4 progressive disease. Thirteen of these complete responses were assessed at second-look procedures and the remainder based on clinical and radiological assessment. Six second-look laparoscopies and 12 second-look laparotomies were carried out on patients in clinical complete remission, with no evidence of disease found in 13, a partial response in 2, static disease in 2 and progressive disease in one. Further tumour debulking was carried out in 4 patients.

*Survival*

Twenty patients have died and a further 3 patients have relapsed but remain alive at 27, 36 and 37 months. One patient died of a pulmonary embolism, one of progressive cachexia shortly after a third laparotomy which showed intra-abdominal fibrosis but no evidence of malignancy, and the remaining 18 patients of progressive malignancy. Second-line cytotoxic chemotherapy was given to 12 patients, and only one achieved a partial response.

The median survival of the entire group (Figure 1) has not been reached at a median follow-up of 2 years and the actuarial survival at that time is 60%. The 9 patients with Stage IV disease had a median survival of 12 months. The median survival of the patients not achieving complete response was also 12 months, whereas of those 31 patients achieving a clinical or pathological complete response only 4 have relapsed within 2 years. Table II shows the risk of relapse by FIGO stage, residual disease volume, and response.

*Subset analysis*

Twenty-four patients in complete clinical or pathological complete remission proceeded to whole abdominopelvic irradiation. Three further patients with static disease (1 patient) and partial response (2 patients) were also given abdominopelvic irradiation at full dose and died 8, 7 and 6 months later, respectively. Three patients have refused radiotherapy.

Of these 24 patients the actuarial disease free survival at 30 months was 75% (Figure 2), there being 4 deaths at 14, 20, 29 and 30 months. All three relapses were in both the abdomen and pelvis, and the fourth patient had the cachexia and extensive intra-abdominal fibrosis referred to above, with no tumour found at laparotomy just before death. This patient, who presented with Stage IV disease, was the only one out of 10 patients with negative second-look procedures completing abdominopelvic radiotherapy who has died at a median follow-up of 33 months. Five of these 10 patients with negative second look operations had disease > 2 cm left after primary surgery. When the patients with Stage I and II disease are excluded, and the analysis restricted to that subset of 15 patients with Stage III and IV disease with

**Figure 1** Survival curve (*upper*) of all 46 patients with advanced ovarian cancer treated with induction chemotherapy comprising cisplatin and cyclophosphamide, and (*lower*) the 9 patients with FIGO Stage IV ovarian cancer. Only one of these 9 patients achieved a CR and received abdominopelvic radiotherapy.

**Table II** Proportion of patients ($n = 46$) relapsing by stage, residual disease and response.

(a) *Responders*

| | *Clinical assessment* | | | *Pathological assessment* | | |
|---|---|---|---|---|---|---|
| | *CR* | *PR* | *Other* | *CR* | *PR* | *Other* |
| Number relapsed | 7 | 4 | 9 | 3 | 2 | 3 |
| Number at risk | 31 | 4 | 15 | 13 | 2 | 3 |
| % relapsed | 22.5 | 100 | 60 | 23.1 | 100 | 100 |

(b) *Stage/Bulk*

| | *FIGO Stage* | | | | *Residual disease*[a] | | | |
|---|---|---|---|---|---|---|---|---|
| | *I* | *II* | *III* | *IV* | *O* | *X* | *XX* | *XXX* |
| Number relapsed | 0 | 1 | 12 | 7 | 0 | 5 | 11 | 4 |
| Number at risk | 6 | 6 | 25 | 9 | 13 | 12 | 17 | 4 |
| % relapsed | 0 | 16.6 | 48 | 77.8 | 0 | 41 | 65 | 100 |

[a]O = microscopic/none; X = <2 cm; XX = 3–9 cm; XXX = >10 cm.

moderate or poor histological differentiation, the actuarial survival at 2 years was 67% (Figure 3).

The 4 patients in whom gross disease more than 10 cm in diameter was present at the start of chemotherapy were all dead within 12 months, but there was no significant difference in actuarial survival between those debulked to <5 cm and those debulked to <2 cm maximum tumour diameter, although the risk of relapse increased progressively with FIGO stage and pre-chemotherapy residual disease (Table II).

*Toxicity*

Patients generally tolerated the chemotherapy without difficulty and toxicity was not excessive. Doses were reduced below 75% of those calculated for cisplatin in 15% of cases,

**Figure 2** Relapse free survival in the 24 patients with advanced ovarian cancer achieving a complete clinical remission with cisplatin and cyclophosphamide followed by abdominopelvic radiotherapy.

**Figure 3** Relapse free survival in the 15 patients with Stages III and IV ovarian carcinoma and moderate or poor histological differentiation given sequential chemotherapy and radiotherapy.

and for cyclophosphamide in 9% of cases. A delay of 2 weeks or more due to myelosuppression was recorded in only 2 patients. One patient refused treatment after the first cycle. Grade III (WHO) nausea and vomiting and alopecia were universal, and Grade III diarrhoea was seen in 2 patients.

Out of 223 cycles of chemotherapy, the total leucocyte count fell below $1.0 \times 10^9 l^{-1}$ in 10 patients (4.5% of cycles) and a fall below $2.0 \times 10^9 l^{-1}$ was recorded in a further 30 patients (13.5% of cycles). There were transfusions given to 12 patients and no episodes of neutropenic fever. The creatinine clearance fell below 50 ml $min^{-1}$ in 6 patients, and this nephrotoxicity was cumulative, reaching a peak incidence after cycle 4.

Four patients discontinued chemotherapy after the fourth cycle. The radiotherapy was well tolerated by the patients although myelosuppression was seen in most cases. A rapid fall in the total WBC in the first week to around $2 \times 10^9 l^{-1}$ was usually seen, but in most cases it was possible to continue radiotherapy without this fall continuing. Suspension of treatment for two or more weeks was required on account of myelosuppression in five other patients, but in only one of these was treatment discontinued and the pelvic boost omitted on account of thrombocytopenia. The median number of treatment days delayed was 12 (mean 11.2 days, range 9–25 days). There were no episodes of neutropenic fever or bleeding, but one case of WHO Grade III late renal damage, a second of Grade III elevated liver function tests and a third of Grade III gastrointestinal toxicity were recorded. In one case a recto-vaginal fistula developed following abdominal irradiation, and the pelvic boost was omitted. One further patient had extensive intraperitoneal fibrosis as discussed above.

### Discussion

We have shown that a regime consisting of aggressive chemotherapy comprising cisplatin and cyclophosphamide followed by a tumoricidal dose of radiation to the abdomen and pelvis can be tolerated by a high proportion of middle-aged women with carcinoma of the ovary. Myelosuppression was acceptable during both the chemotherapy and radiotherapy components of treatment. The relative lack of toxicity seen with the radiation therapy may be related in part to the absence of adriamycin from the induction regime.

These results show a complete response rate to chemotherapy of 72% which compares favourably with other published regimes. It has been estimated that cisplatin containing combinations prolong the median survival time in advanced ovarian cancer by 6–12 months over those who have never received cisplatin or its analogues (Dembo, 1986), although there is still controversy over the size of the effect. This survival benefit of chemotherapy has been particularly marked in those patients with disease debulked to a maximum residual diameter of 3 cm or less (Ozols & Young, 1984) and it is at this volume that the dose of achievable radiation therapy reaches its optimum (Tubiana, 1983), as the number of residual clonogenic cells is low.

In the present study the 24 patients in complete remission given combined modality treatment (Figure 2) provide evidence that the combined approach of chemotherapy and radiotherapy may have produced a significant cohort of long term survivors, the only death being associated with extensive peritoneal fibrosis with no evidence of recurrent tumour. This is emphasised by the subset analyses of the 10 patients in pathological complete remission, and in the poor risk intra-abdominal disease patients (Figure 3) given both treatments. A negative second-look procedure confirms the first requirement has been met for a treatment approach designed to achieve prolonged survival, but almost certainly further consolidation chemotherapy or radiation therapy is required to improve the long term results in this disease (Sutton *et al.*, 1986; Cain *et al.*, 1986). In the 3 patients given abdomino-pelvic radiotherapy where macroscopic disease was present, the short survival would indicate this treatment was of little benefit in this group.

The lack of an effect of moderate tumour bulk on survival either at presentation or after surgery may be further evidence that the irradiation is providing a consolidation effect to the chemotherapy, as in previous studies with surgery and chemotherapy alone, bulk has been an important variable in determining outcome (Williams *et al.*, 1982). However, the 9 patients with stage IV disease have shown a particularly poor survival, as did those 4 in whom no debulking was possible, and in the entire group FIGO stage and residual disease were related to relapse. It may be that the critical site of tumour (e.g. in bowel wall or liver) is more important than bulk: alternatively this intensive treatment approach has overcome an adverse factor applicable only to these earlier studies.

The present series compares favourably in outcome to other studies of sequential chemotherapy and radiotherapy. Hainsworth *et al.* (1983) found that 15 out of 17 patients given sub-optimal doses of chemotherapy and radiotherapy relapsed within a median of 8 months. Rustin *et al.* (1987) employed a similar approach, but omitted the boost to the pelvis, and in 27 patients with Stage III disease, 8 of whom were in pathological complete remission, found a median progression free survival of 19 months. In the present study the restriction of the 2 drug combination to 5 cycles and the low fraction size of radiotherapy may have contributed to the improved results.

This aggressive approach deserves further evaluation in adequately debulked patients without visceral involvement. It is clear that the selection of an adequate dose and schedule of chemotherapy (Piccart *et al.*, 1987) and of radiation therapy (Dembo *et al.*, 1979) is vital to achieve disease control without excessive toxicity. However, one third of patients may not reach this stage of treatment on account of failure to achieve response to chemotherapy or refusal of patients to accept the complete course of intensive treatment. Patients with poor prognostic factors, such as Stage IV disease, require more intensive induction chemotherapy. In those patients responding to chemotherapy the abdomino-pelvic radiotherapy given in the present study needs to be compared in a randomised trial to continuing chemotherapy or no further therapy.

The authors would like to thank Mr B. Alderman, Mr A. Murray, Mr S.J. Leinster, Mr W. Gault and Mr DeBoer for referring patients to this study, and Miss J.L. Murray for expert typing of the manuscript.

### References

ARMITAGE, P. (1971). *Statistical methods in medical research.* Halstead Press: London.

CAIN, J.M., SAIGO, P.E., PIERCE, V.K. & 6 others (1986). A review of second look laparotomy for ovarian cancer. *Gynaecol. Oncol.*, **23**, 14.

DEMBO, A.J. (1986). Controversy over combination chemotherapy in advanced ovarian cancer: What we learn from reports of matured data, *J. Clin. Oncol.*, **4**, 1573.

DEMBO, A.J. & BUSH, R.S. (1983). Radiation therapy of ovarian cancer. In *Gynaecological Malignancy*, Griffiths, C.T. & Fuller, A.F. Jr. (eds) p. 263. Martinus Nijhoff: The Hague.

DEMBO, A.J., BUSH, R.S., BEALE, F.A. & 4 others (1979). Ovarian carcinoma: Improved survival following abdomino pelvic irradiation in patients with a complete pelvic operation *Am. J. Obstet. Gynecol.*, **134**, 793.

EDMONSON, J.H., McCORMACK, G.W., FLEMING, T.R. & 8 others (1985). Comparison of cyclophosphamide and cisplatin versus hexamethylmelamine, cyclophosphamide, doxorubicin and cisplatin in combination as initial chemotherapy for Stages III and IV ovarian carcinoma. *Cancer Treat. Rep.*, **69**, 1243.

GERSHENSON, D.M., COPELAND, L.J., WHARTON, J.T. & 4 others (1985). Prognosis of surgically determined complete responders in advanced ovarian cancer. *Cancer*, **55**, 1129.

GREEN, J.A. & YOUNG, R.C. (1983). Gynaecological tumours. In *Cancer Chemotherapy Annual* 5, Pinedo, H.M. (ed) p. 369.

GREINER, R., GOLDHIRSCH, A., DAVIS, B.W. & 7 others (1984). Whole abdomen radiation in patients with advanced ovarian carcinoma after surgery, chemotherapy and second-look laparotomy. *J. Cancer Res. Clin. Oncol.*, **107**, 94.

GRIFFITHS, C.T., PARKER, L.M. & FULLER, R.F. (1979). Role of cytoreductive surgical treatment in the management of advanced ovarian cancer. *Cancer Treat. Rep.*, **63**, 235.

HACKER, N.F., BEREK, J.S., BURNISON, C.M., HEINTZ, P.M., JUILLARD, G.J. & LAGASSE, L.D. (1985). Whole abdominal irradiation and salvage therapy for epithelial ovarian cancer. *Obstet. Gynaecol.*, **65**, 60.

HACKER, W.F., BEREK, J.S., LAGASSE, L.D., NIEBERG, R.K. & ELASHOFF, R.M. (1983). Primary cytoreductive surgery for epithelial cancer, *Obstet. Gynaecol.*, **61**, 413.

HAINSWORTH, J.D. MALCOLM, A., JOHNSON, D.H., BURNETT, L.S., JONES, H.W. & GRECO, F.A. (1983). Advanced mimimal residual ovarian carcinoma. Abdominopelvic irradiation following combination chemotherapy. *Obstet. Gynaecol.*, **61**, 619.

MARTINEZ, A., SCHRAY, M., HOWES, A. & BAGSHAWE, M.A. (1985). Post operative radiation therapy for epithelial ovarian cancer. The curative role based on a 24 year experience. *J. Clin. Oncol.*, **3**, 901.

MUIR, C.S. & NECTOUSE, J. (1978). Ovarian cancer – some epidemiological features. *WHO Statistical Reports*, **31**, 51.

NEIJT, J.P., TEN BOKKEL HUININK, W.W., VAN DEN BURG, M.E.L. & 7 others (1985). Combination chemotherapy with CHAP-5 and cisplatin in advanced ovarian carcinoma: A randomised trial of the Netherlands Joint Study Group for ovarian cancer. *Proc. Am. Soc. Clin. Oncol.*, **1**, 114 (*abstract*).

NEIJT, J.P., VAN DER BURG, M.E.L., VRIESENDORP, R. & 8 others (1984). Randomised trial comparing two combination chemotherapy regimens (Hexa-CAF *vs.* CHAP-5) in advanced ovarian carcinoma. *Lancet*, **ii**, 594.

ONNIS, A. & MAGGION, T. (1984). Repetitive debulking surgery as adjuvant to chemotherapy in advanced epithelial ovarian cancer. *Clin. Exp. Obstet. Gynaecol.*, **11**, 21.

OZOLS, R.F. & YOUNG, R.C. (1984). Chemotherapy of ovarian cancer. *Semin. Oncol.*, **11**, 251.

PICCART, M.J., SPEYER, J.L., WERNZ, J.C. & 6 others (1987). Advanced ovarian cancer: Three-year results of a 6–8 month, 2 drug cisplatin containing regime. *Eur. J. Cancer Clin. Oncol.*, **23**, 631.

RICHARDSON, G.S., SCULLY, R.E., NIKRIN, N. & NELSON, J.H. (1985*a*). Common epithelial cancer of the ovary. *New Engl. J. Med.*, **312**, 415.

RICHARDSON, G.S., SCULLY, R.E., NIKRIN, N. & NELSON, J.H. (1985*b*). Common epithelial cancer of the ovary. *New Engl. J. Med.*, **312**, 474.

RIZEL, S., BIRAN, S., ANTEBY, S.O., BRUFMAN, G., SULKES, A. & MILCHWIDSKY, A. (1985). Combined modality treatment for Stage III ovarian carcinoma. *Radiother. Oncol.*, **3**, 237.

RUSTIN, G.J.S., MINTON, M., SOUTHCOTT, B. & 4 others (1987). Surgery, chemotherapy and whole abdominal radiotherapy in the management of advanced ovarian carcinoma. *Clin. Radiol.*, **38**, 269.

SMITH, J.P., RUTLEDGE, P.N. & DELCLOS, L. (1975). Post operative treatment of early cancer of the ovary: A randomised trial between post operative irradiation and chemotherapy. *Natl Cancer Inst. Monog.*, **42**, 149.

STEINER, M., RUBINOV, R., BOROVIK COHEN, Y. & ROBINSON, E. (1985). Multimodal approach in the treatment of advanced ovarian carcinoma. *Cancer*, **55**, 2748.

SUTTON, G.P., STEHMAN, F.B., EHRLICK, C.E., EINHORN, L.H., ROTH, L.M. & BLESSING, J.A. (1986). Seven year follow-up of patients receiving cisplatin, adriamycin and cyclophosphamide (PAC) chemotherapy for Stages III and IV epithelial ovarian carcinoma. *Proc. Am. Assoc. Clin. Oncol.*, **5**, 120 (*abstract*).

TUBIANA, M. (1983). The causes of clinical radioresistance. In *The Biological Basis of Radiotherapy*, Steel, G.G. *et al.* (eds) p. 13. Elsevier: Oxford.

WILLIAMS, C.J., MEAD, B., ARNOLD, A., GREEN, J.A., BUCHANAN, R. & WHITEHOUSE, J.M.A. (1982). Initial experience with cisplatin, adriamycin and cyclophosphamide in the management of advanced ovarian carcinoma. *Cancer*, **49**, 1778.

YOUNG, R.C. (1979). Gynaecologic malignancies. In *Cancer Chemotherapy Annual* 1, Pinedo, H.M. (ed) p. 340.